# Lignin Nanoparticles: A Promising Tool to Improve Maize Physiological, Biochemical, and Chemical Traits

**DOI:** 10.3390/nano11040846

**Published:** 2021-03-26

**Authors:** Daniele Del Buono, Francesca Luzi, Debora Puglia

**Affiliations:** 1 Dipartimento di Scienze Agrarie, Alimentari e Ambientali, Università degli Studi di Perugia, Borgo XX Giugno 74, 06121 Perugia, Italy; daniele.delbuono@unipg.it; 2 Department of Civil and Environmental Engineering, University of Perugia, Strada di Pentima 4, 05100 Perugia, Italy; francesca.luzi@unipg.it

**Keywords:** lignin nanoparticles, maize, biostimulatory action, germination, plant performance

## Abstract

Lignin, and its derivatives, are the subject of current research for the exciting properties shown by this biomass. Particularly attractive are lignin nanoparticles for their eco- and biocompatibility compared to other nanomaterials. In this context, the effect of nanostructured lignin microparticles (LNP), obtained from alkaline lignin by acid treatment, on maize plants was investigated. To this end, maize seeds were primed with LNP at five concentrations: 80 mg L^−1^ (T80), 312 mg L^−1^ (T312), 1250 mg L^−1^ (T1250), 5000 mg L^−1^ (T5000) and 20,000 mg L^−1^ (T20000). Concerning the dose applied, LNP prompted positive effects on the first stages of maize development (germination and radicle length). Furthermore, the study of plant growth, biochemical and chemical parameters on the developed plants indicated that concerning the dose applied. LNP stimulated beneficial effects on the seedlings (fresh weight and length of shoots and roots). Besides, specific treatments increased the content of chlorophyll (a and b), carotenoid, and anthocyanin. Finally, the soluble protein content showed a positive trend in response to specific dosages. These effects are significant, given the essential biological function performed by these biomolecules. In conclusion, this research indicates as the nanostructured lignin microparticles can be used, at appropriate dosages, to induce positive biological responses in maize. This beneficial action deserves attention as it candidates LNP for biostimulating a crop through seed priming.

## 1. Introduction

Lignin is one of the most abundant biomasses on earth. In terms of quantity, lignin is second after cellulose, being present in up to 30% of woody biomass [1]. The macromolecular structure of lignin is incredibly complex, and it strongly depends on the plant species from which it derives. In general, lignin is an intricate network of aromatic structures resulting from the radical bonding of phenylpropanoid monomers [2]. However, three types of phenols are the basic units that compose lignin: p-hydroxyphenyl (from coumaryl alcohol, also called lignin H), guaiacyl (from coniferyl alcohol with a methoxy group, also called lignin G), or syringyl (from synaphyl alcohol with two methoxyl groups, also called lignin S) [2]. The relative amount of these phenolic monomer units varies in close correlation with the species botanical origin and the methods applied for lignin extraction [3]. Moreover, these aspects control the heterogeneity and properties of this macromolecule [3]. 

Lignin is increasingly considered a material of interest for its potential to produce fuels and the development of innovative non-toxic, and environmentally friendly materials [4]. However, some lignin characteristics such as large particle size, irregularity, and heterogeneity limit its potential applications [2]. Despite this, lignin can undergo chemical reactions to insert new functional groups in its structure or obtain nanoparticles to improve the crucial biomass characteristics mentioned above [4]. 

In this context, due to the growing interest in nanotechnology [5,6], the possibility of converting lignin into nanoparticles (LNP) has been increasingly studied. In particular, the biodegradable nature of LNP makes them particularly attractive as more significantly eco- and biocompatible than other nanomaterials [7]. For instance, lignin nanoparticles have found wide application in cosmetics, in the preparation of nanocomposites, in medicines or as nano-precursors [1]. Of particular interest are the antioxidant properties of LNP due to their stable organic radicals in the conjugated π-electrons of the phenolic aromatic structures [8]. This activity enables lignin to absorb and inactivate reactive oxygen species (ROS) and, in particular, those in the radical form [8]. Moreover, the greater or lesser effectiveness in removing radicals is related to the species botanical origin, with S and G units being the main responsible for lignin antioxidant activity [8].

Another promising application that involves lignin, as nanoparticles or not, is its use as a valuable macromolecule for environmental remediation [9]. To this end, lignin has been successfully employed for removing toxic substances from the environment (soil and water), and in particular heavy metals [9,10,11]. In addition, agriculture is trying to implement this type of nanoparticles for the controlled release of agrochemicals and fertilizers [4,12,13,14]. Some studies report the utilization of nanoparticles to overcome nutrient deficiencies [15,16] as carriers for herbicides [17,18] and fungicides [19]. Targeted and sustained delivery, efficient uptake of nutrients, and limited environmental impacts are the main positive points of using nano- and micro-carriers in agriculture [20,21]. Specific works dealt with the possible application of lignin at the nanoscale as a carrier [22] and encapsulating agent for bioactive additives [23,24]. Besides, lignin nanoparticles may find use in restoring the humic fraction of soil [4,25]. Furthermore, there is the possibility of using lignin in agriculture as a material that could positively affect the development and physiological and biochemical traits of crops, acting as a biostimulant. Biostimulants are a broad family of substances obtained from a wide range of raw materials with the sole purpose of producing beneficial direct or indirect effects on crops [13]. These materials act in crops by improving the nutrient acquisition and use, resistance to abiotic and biotic stress, quality aspects, and productivity [26,27]. 

In this context, some recent studies conducted directly on lignin and some derivatives have shown positive and encouraging effects of these materials on tomato, watercress, chicory, and maize [28,29,30]. It was found that water-soluble humic-like lignins improved plant and seed development by directly acting as gibberellin (GA) molecules or positively perturbing GA-related hormonal balances. These effects support lignin hydrophilicity as the main characteristic responsible for the effective release of bioactive molecules. To this end, some authors found beneficial effects on plant germination, seed development, hormonal balance, and growth, depending on the lignin botanical origin [28,29], surface chemistry (changes in amphiphilic groups) and size (aggregation patterns) [31]. Therefore, these studies consider the candidate lignin and its derivatives as an enhancer of seed germination and potential biostimulant to prompt beneficial effects on crops.

However, despite the experimental pieces of evidence mentioned earlier on the lignin effectiveness in acting as a plant biostimulant, the investigations of lignin nanoparticles impact on crops remain entirely unexplored and an open field. The interest in studying LNP and their biological effect on plants stems from the fact that, as with other nanomaterials, nanoparticles can exhibit interesting biological properties. In particular, such an effect could be related to their size and morphological characteristics [32]. The interest in this nanoparticle system also derives from the possibility of finding new valuable uses of lignin, which otherwise must be disposed of because it is considered waste.

Therefore, this research aimed to investigate the possibility of inducing some beneficial effect in maize using lignin nanoparticles. For this purpose, LNP was obtained by acid treatment of an alkali lignin precursor. Then, the effect of LNP on maize was investigated over a wide range of concentrations. Some physiological and biochemical parameters were monitored to clarify the effects of LNP on the crop in question.

## 2. Materials and Methods

### 2.1. Materials

Alkali lignin was used as the raw materials for the preparation of nanostructured lignin microparticles (LNP). Alkali lignin hydroxide, hydrochloric acid (HCl, 35%), ethylene glycol (99.8%), potassium bromide (KBr), sodium hypochlorite (NaClO, 98%), calcium nitrate tetrahydrate (Ca(NO_3_)_2_ × 4H_2_O, 98%), magnesium sulphate heptahydrate (MgSO_4_ × 7H_2_O, 98%), potassium sulphate (K_2_SO_4_, 98%), potassium chloride (KCl, 99%), potassium phosphate monobasic (KH_2_PO_4_, 99%), boric acid (H_3_BO_3_, 99.5%), magnesium sulphate monohydrate (MnSO_4_ × H_2_O, 98%), copper sulphate (CuSO_4_, 99%), zinc sulphate heptahydrate (ZnSO_4_ × 7H_2_O, 98%), ammonium molybdate tetrahydrate ((NH_4_)_6_Mo_7_O_24_ × 4H_2_O, 99%), ethylenediaminetetraacetic acid ferric sodium salt (Fe-EDTA), acetone (99.8%), tris(hydroxymethyl)aminomethane (Tris, 99.8%), bovine serum albumin (BSA, 98%) were provided by Merck Life Science S.r.l. (Milan, Italy). All of the chemicals were used as received without additional purification.

### 2.2. Synthesis of Nanostructured Lignin Microparticles

LNP suspension was obtained from alkali lignin by hydrochloric acid treatment based on the procedures reported in the literature [33,34,35]. 4% (*w*/*v*) of alkali lignin in ethylene glycol was maintained under a stirrer for 2 h at 35 °C. Afterwards, HCl (8 mL, 0.25 M) was mildly added to the solution at a rate of 3–4 drops/min. After that, the suspension was stirred again for 2 h. The product was filtered to eliminate soluble impurities from lignin. The solution was then dialyzed against deionized water up to neutrality to obtain the LNP suspension.

### 2.3. Characterization of Nanostructured Lignin Microparticles

Nanostructured lignin microparticles were morphologically and chemically investigated. The morphology of the nanoparticles was analyzed by using scanning electron microscopy (FESEM, Supra 25-Zeiss, Oberkochen, Germany). A small drop of lignin nanoparticle water suspension was deposited on silicon substrates, air-dried for 24 h, gold coated by using an ion sputter coater, and observed by FESEM and field emission gun operated at 5 kV.

Fourier infrared (FT-IR) spectra of alkali lignin and lignin nanoparticles were recorded using a Jasco FT-IR 615 spectrometer (Jasco Corporation, Tokyo, Japan) in the 4000–600 cm^−1^ range, in transmission mode. The lignocellulosic materials were analyzed using KBr discs prepared by using pulverized natural materials and KBr powder.

### 2.4. Plant Growth, Treatments, Germination, Shoot and Root Length

Maize (cv Belgrano) seeds were sterilized for 3 min with a solution of NaClO (0.25%, *w*/*v*). Seeds were then rinsed several times with distilled water. To prime maize seeds, five different LNP suspensions were prepared at the concentrations of 80 mg L^−1^ (T80), 312 mg L^−1^ (T312), 1250 mg L^−1^ (T1250), 5000 mg L^−1^ (T5000), and 20,000 mg L^−1^ (T20000). Successively, the seeds were immersed in 10 mL of these solutions for 8 h for seed priming in slow agitation. Seeds were then positioned on paper in Petri dishes (10 seeds/plate) and added with 10 mL of distilled water. These samples were covered and placed into a growth chamber in the dark (22 ± 2 °C). Controls seed were obtained for priming them with distilled water. Four days after sowing (DAS), seed germination was recorded, while at 5 DAS, radicle lengths were measured. 

After that, seedlings were transferred into hydroponic solutions containing a nutrient solution 2 mmol L^−1^ Ca (NO_3_)_2_ × 4H_2_O, 0.5 mmol L^−1^ MgSO_4_ × 7H_2_O, 0.7 mmol L^−1^ K_2_SO_4_, 0.1 mmol L^−1^ KCl, 0.1 mmol L^−1^ KH_2_PO_4_, 1 μmol L^−1^ H_3_BO_3_, 0.5 μmol L^−1^ MnSO_4_ × H_2_O, 0.5 μmol L^−1^ CuSO_4_, 0.5 μmol L^−1^ ZnSO_4_ × 7H_2_O, 0.01 μmol L^−1^ (NH_4_)_6_Mo_7_O_24_ × 4H_2_O, and 100 μmol L^−1^ Fe-EDTA. 

At the third leaf stage, namely 2 weeks after sowing (14 DAS), plants were harvested and subjected to the following determinations.

### 2.5. Chlorophyll, Carotenoids, Anthocyanin, and Soluble Protein Determinations

The contents of chlorophyll a and b and carotenoids were assessed in maize samples subjected to the different treatments, as described above, and collected at 14 DAS. In particular, 1.0 g of leaf samples was extracted with 85% acetone in water (*v*/*v*), with a pestle in a mortar using quartz sand. After that, the suspensions were filtered, and the absorbance was recorded at 452.5, 644, and 663 nm. The pigment content was determined according to Venkatachalam et al. [36].

Furthermore, 0.5 g of the harvested maize shoots were extracted with ethanol (95%) with pestle and mortar to evaluate the anthocyanin content. The resulting suspension was filtered and centrifuged at 7000 rpm for 20 min at 8 °C. Finally, the anthocyanin content was determined spectrophotometrically at 535 and 650 nm, according to Lichtenthaler and Buschmann [37].

About 0.5 g of leaf samples were homogenized in 5 mL of 0.1 M Tris-HCl buffer (pH 7.5) using a cold mortar and pestle. The extract was centrifuged at 10,000 rpm for 15 min at 4 °C. Then, the protein content was estimated in the supernatant, according to Bradford [38] using bovine serum albumin (BSA) as standard. 

### 2.6. Statistical Analysis 

Each value reported represents the mean of the data from three independent experiments on at least three biological replicates per experiment. Statistical analysis of the data was performed in ANOVA mode by analyzing the variance with Duncan’s test at *p* < 0.05. The *R* statistical environment was used to perform the statistical analysis [39].

## 3. Results

### 3.1. Characterization of Nanostructured Lignin Microparticles

**Figure 1:**
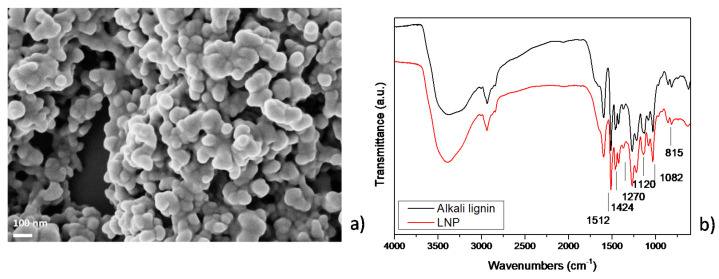
(**a**) FESEM image of lignin nanoparticles (LNPs) and (**b**) FT-IR spectra of micrometric lignin and LNPs in the ranges of 4000–600 cm^−1^.

Results of FESEM analysis of LNP (Figure 1a) showed that the nanoparticles have diameters in the range of 50 ± 20 nm [35,40] and appear in the form of aggregates: this phenomenon is related to the hydrodynamic radius of colloidal lignin in aqueous suspension, that can be influenced by different parameters, such as temperature, pH [41,42], and lignin concentration [43].

FTIR analysis of pristine alkali lignin and LNP was also conducted (Figure 1b). The results of the analysis showed that alkali lignin has a broad band at 3600–3300 cm^−1^, corresponding to hydroxyl groups in phenolic and carboxylic acids and several bands with variable intensity in the fingerprint region (1900 to 800 cm^−1^). In this region, the main features appear at 1600–1500 cm^−1^ (C=C skeletal vibrations), 1460 cm^−1^ (C-H deformation combined with aromatic ring vibrations), 1270 cm^−1^ (C=O stretch), 1218 cm^−1^ (C-C, C-O and C=O stretching), 1140 cm^−1^ (C-H in plane deformation), 1033 cm^−1^ (complex vibration associated with the C-O, C-C stretching and C-OH bending in polysaccharides), and 856 and 815 cm^−1^ (C-H out-of-plane deformations). The band appearing at 620 cm^−1^ is assigned to the sulfonic groups (S-O stretching vibration) formed from sodium sulfite reaction with the secondary OH of the aliphatic side chain of lignins [44]. Changes in FTIR spectrum of LNP can be detected at 1512 and 1424 cm^−1^, attributed to the aromatic ring vibrations of phenylpropanoid (C9) skeleton of lignin. Also, the peak at 1670 cm^−1^, related to the ring-conjugated C=O stretch of coniferaldehyde/sinapaldehyde, resulted in a decrease in intensity. The bands around 2936 and 2840 cm^−1^ can be attributed to C-H stretch present in methyl, methylene, and methoxyl groups in LNP. The relative intensity of the methoxyl group band was higher in LNP when compared to alkali lignin, specifying that LNP has more syringyl (S) units over guaiacyl (G) units. Further, bands detected at 1270 and 815 cm^−1^ correspond to C=O stretch in guaiacyl unit and C-H out-of-plane vibrations in 2, 5, and 6 positions of guaiacyl units [45]. Furthermore, the spectrum of LNP shows intensity variation for the band at 1120 cm^−1^ and 1082 cm^−1^, assigned, respectively, to condensed aromatic units [46] and C-O stretch of secondary alcohols and aliphatic ethers [47]. During the acid treatment procedures, aromatic rings of lignin are stable, whereas the methoxyl group (−OCH_3_) in the aromatic ring and C3 content (including Ar=O and Ar-C=O groups) increased, as already observed by NMR observation [1,34].

### 3.2. Effects of Nanostructured Lignin Microparticles on Seed Germination and Plant Growth

**Figure 2:**
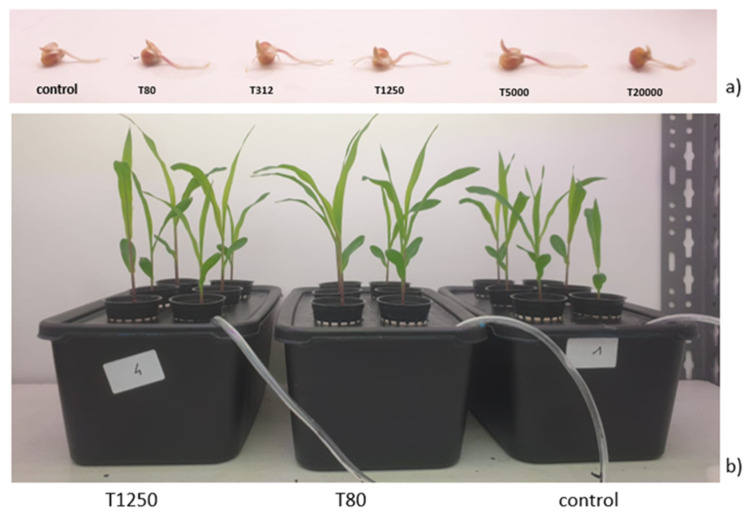
Representative seeds at 4 days after treatment with the various LNP concentrations (T80, T312, T1250, and T5000 refer to LNP concentration applied to seed for priming (**a**); representative samples at 14 days after the treatment with LNP (T80 and T1250 refer to the LNP concentrations used for seed priming (**b**).

The effect of the treatments with the five concentrations of LNP on maize germination was recorded after four days and compared with control seeds (Table 1 and Figure 2a).

T312, T1250, and T5000 significantly stimulated maize germination, while T80 did not affect this parameter (Table 1). On the other hand, the treatment T20000 significantly reduced the maize capacity to germinate. Radicle length was affected by the treatments (Table 1). In particular, except for T20000, all the LNP treatments determined increases in the radicle lengths. 

Since T20000 exerted an inhibitory effect on maize germination, this dose was not included in the subsequent investigations. 

**Figure 3:**
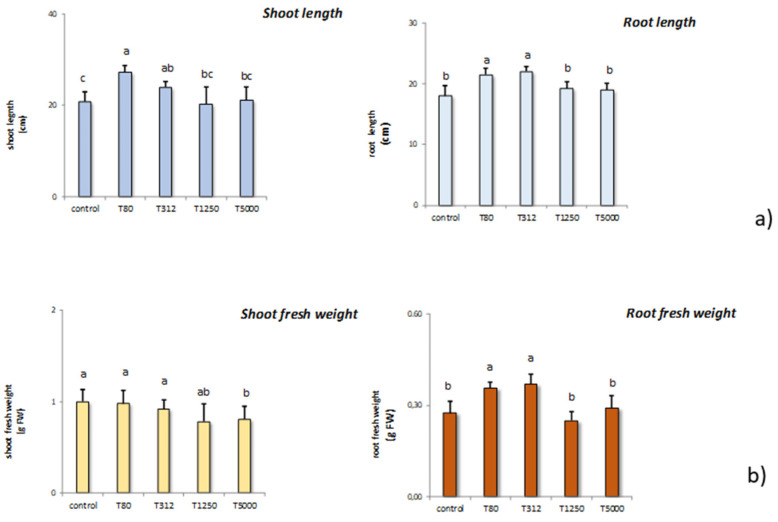
Effect of the treatment with LNP on the shoot and root length of maize samples compared to the untreated controls (T80, T312, T1250, and T5000 refer to LNP concentration applied to seed for priming (**a**); effects of the treatments with LNP on the shoot and root weight of maize seedlings compared to the untreated control (**b**). The values were recorded on seedlings at 14 DAS. Letters in the figure, if different, indicate statistically significant differences for *p* < 0.05 between treatments.

LNP concentrations T80, T312, T1250, and T5000 differently affected shoot and root length in maize (Figure 3a). As far as shoot length is concerned, the treatments capable of stimulating shoot length were T80 and T312. The other treatments were ineffective in affecting the shoot length. Also, in the case of root length, T80 and T312 effectively increased this parameter compared to the control samples. The others were again effective in influencing root length. 

Subsequently, we monitored the different treatment solutions on shoot and root fresh weight in maize (Figure 3b). In general, all the treatments did not influence shoot fresh weight. The only exception was T5000 that decreased these parameters significantly if compared to the control samples. On the contrary, root fresh weight was stimulated by T80 and T312, while the other treatments were ineffective compared to the untreated controls. 

### 3.3. Chlorophyll, Carotenoid, Anthocyanin, and Soluble Protein Content Showed by Maize Treated with Lignin Nanoparticles

**Figure 4:**
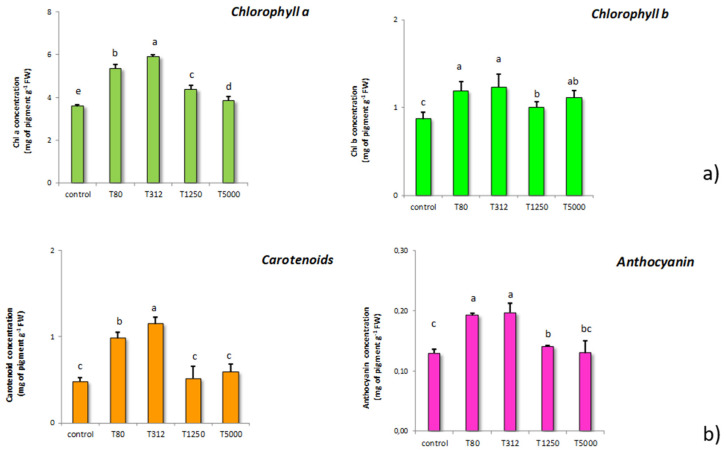
Effects on chlorophyll a and b content determined in maize seeds treated with LNP compared to control samples (T80, T312, T1250, and T5000 refer to LNP concentration applied to seed for priming) (**a**); content of carotenoid and anthocyanin in maize subjected to the treatments with LNP compared to the control samples (**b**). The values were recorded on seedlings at 14 DAS. Letters in the figure, if different, indicate statistically significant differences for *p* < 0.05 between treatments.

The chlorophyll a and b content was ascertained in the samples exposed to the different LNP concentrations (Figure 4a). T80 and T312 strongly increased the content of these pigments. In particular, for these two concentrations, the increases of chlorophyll a exceeded 50%, while the increases in chlorophyll b were around 40%. The other LNP concentrations led to increases in chlorophyll a and b, but these were much smaller than those found for the other doses. Concerning chlorophyll b, all treatments increased the content of this pigment (Figure 2). In particular, T80, T312, and T5000 provoked the highest increases if compared to the control samples.

Concerning carotenoids, Figure 4b illustrates the results obtained. Significant increases in carotenoids were found in samples treated with T80 and T312, while the other LNP dosages did not affect the carotenoid content. With regard to anthocyanin, the treatments with T80 and T312 determined substantial increases in its content. Additionally, even T1250 and T5000 increased the anthocyanin content compared to the control samples; nonetheless, the magnitude of these increases was significantly lower than that induced by the T80 and T312.

**Figure 5:**
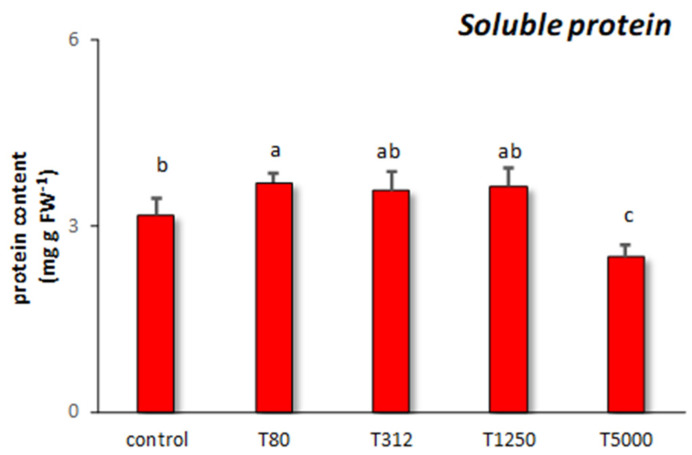
Soluble protein content found in maize samples treated with different concentrations of LNP compared to the control samples (T80, T312, T1250, and T5000 refer to LNP concentration applied to seed for priming). The values were recorded on seedlings at 14 DAS. Letters in the figure, if different, indicate statistically significant differences for *p* < 0.05 between treatments.

As for soluble protein, T80 determined the highest increase in its content (Figure 5). The other assays had no significant effect on soluble protein compared to the control samples.

## 4. Discussion

In recent years there has been growing interest in nanotechnologies because of the wide variety of possible applications in different scientific and technological fields. This interest has led to plant biomasses being considered substrates to synthesize various nanomaterials [2]. To this end, materials containing lignin have been considered as excellent precursors to be used, after isolation of this macromolecule, to obtain nanoparticles to apply in various fields. For instance, lignin nanoparticles have been used for their antioxidant and antimicrobial properties, for their protective capacity against UV radiation, for drugs delivery, environmental remediation, or for making hybrid nanocomposites [48,49,50,51,52]. However, little attention has been paid to using this biomass in nanoparticle form for agricultural purposes, other than as a support for released pesticide control [2]. In particular, very insufficient attention has been devoted to studying lignin biological properties and its derivative on crops, although recent research has highlighted its potential to stimulate crops [53]. 

Relatively to lignin nanoparticles, to the best of our knowledge, no information is available on their effect and use to stimulate biological responses in crops. Therefore, this study aimed at this aspect by investigating the effects of different concentrations of LNP on maize when administered via seed priming. This way of treating plant can affect germination and the early stages of plant development [54]. 

Four of the five concentrations of LNP used in this study had an inductive effect on germination (Table 1), while the highest dose (T20000) had an inhibitory effect on seed development. Positive effects on seed germination have already been found for lignin extracts obtained from Giant Reed [28]. This stimulatory action was explained based on the content of this matrix. In particular, phenol-rich materials, such as lignin, may stimulate metabolic processes leading to improved seed development [28]. This effect can be attributed to a hormone-like action that lignin, and the phenolic compounds it contains, can exert on the early seed’s biochemical activities [39]. Indeed, lignin can show a gibberellin-like action that results in a beneficial effect, thus influencing the seed’s hormonal status and physiological mechanisms underlying its development [28]. Furthermore, it has been hypothesized that lignin could increase seed germination for its capacity to act directly on mitotic activity [55]. In a recent paper, Falsini et al. [56] postulated beneficial effects on plants due to many hydrophilic groups in the lignin chemical structure. In particular, these groups, located on the external surface of nanoparticles in an aqueous environment, could positively affect the processes related to plant germination and growth by increasing the water availability. In our specific case, we have already observed that the fractionation of pristine lignin reduced the presence of aromatic and phenolic, implying that the aromatic rings were possibly oxidized to quinones rings. Additionally, the decrease in peaks assigned to the aldehyde during the acid treatment procedure demonstrated that some ester bonds or carboxyl groups formed [1]. The acid hydrolysis treatment on the pristine lignin also caused a decline of the average molar mass, inducing a broader polydispersity index (1.42) when compared with pristine microlignin (1.34), which suggests an abundance of lower molecular weight particles [1]. On the other hand, some studies showed that lignin could not show stimulatory effects on seed germination [29,57]. Therefore, the effect on this critical phase of plant development is related to the type, composition, and treatments which lignin undergoes. On this account, our results indicate that the LNP, given their structural properties and small size (50 ± 20 nm) [1], may have been absorbed by seeds more efficiently than other raw lignin precursors, thus playing a significant biological role in the germination of maize seeds. As for T20000, the excessive concentration of LNP had a toxic effect that inhibited seed development. This adverse action is due to the high concentration of lignin-phenolic compounds. Likewise, it has been documented that certain compounds can impact the first stages of plant development, for the particular sensitivity showed by seeds to excessive amounts of polyphenols [58]. Finally, it is to note that effective seed priming can make young plantlets more capable of responding to environmental stimuli and stress [59]. 

Concerning radicle length (Table 1), all treatments stimulated maize seed development five days after sowing, with the sole exception of the more concentrated dosage (T20000). This inductive effect results from the ability of LNP to stimulate seed germination. In fact, the improvement in radicle development is even considered hormonal, and it results from lignin composition and structure [60]. In particular, lignin humic-like activity can have a decisive influence on germination and affect the radicle length [61]. These positive effects on the early developmental stages are relevant since seed priming’s effectiveness can influence the plant’s subsequent development [49,62]. 

The maize seedlings were monitored for two weeks after sowing, recording shoot and root lengths and weights (Figure 3 and Figure 4). In general, the two lowest LNP doses, T80 and T312, showed a positive effect on the ability of maize to produce biomass (Figure 3 and Figure 4). 

Differently, the other treatments were ineffective or even interfered negatively with this aspect. Increases in plant biomass have been documented in response to the treatment with lignin and phenolic substances [63,64]. In particular, the syringyl phenols, which can act in showing a gibberellin-like activity, can induce seedling development [65]. The nanoparticles used in this study show high syringyl groups content [1]. Therefore, the increases in plant biomass (Figure 3 and Figure 4) are in line with Nardi et al. [65] and Savy et al. [60] who found, that lignin derivatives positively influenced maize development, despite not affecting the germination. Positive effects on plant biomass can also be ascribed to the presence of guaiacyl and p-hydroxyphenyl groups into the lignin [53]. In particular, these two components can improve root system development and biomass [66]. Besides, substances containing phenolic compounds positively affect plant growth by improving the uptake of essential macronutrients in seedlings. In this sense, Ertani et al. [64] showed that phenolic substances by stimulating the root system improved the maize assimilation of N, P, Ca, K, and Mg. 

As for the chlorophyll a and b contents recorded in maize samples treated with the various LNP concentrations, different effects were recorded (Figure 5). Significant increases in maize chlorophyll content were found for all the treatments. The inductive effects exerted by LNP on chlorophyll a and b results from a stimulatory action on their biosynthesis. Some phenolic compounds can increase the content of the pigments mentioned above, owing to their capacity to improve plant nutrition, particularly nitrogen assimilation [67]. Furthermore, it should be noted that a good root development, as prompted by T80 and T312, can justify the highest content of chlorophyll a and b since a better root system can allow maize to increase its capacity to take up nutrient from the growth media [68]. Finally, higher chlorophyll concentrations are considered a normal physiological adaptation to external stimuli, which improve the crop capacity to harvest light in photosystems [69]. 

It is necessary to note that the ratio chlorophyll a/b ratio increased for T80, T312, and T1250, while it decreased for T5000 compared to controls (data not reported). This effect resulted from the substantial increase in chlorophyll b caused by the latter treatment, which was not supported by an increase in chlorophyll a. The ratio chlorophyll a/chlorophyll b has been proposed as a parameter to evaluate crops response to environmental adversities, diseases, and stresses [70]. In particular, some stressors can provoke the conversion of the chlorophyll a to b via the enzyme activity of Chl a oxygenase [71]. Thus, the increase in chlorophyll b, not counterbalanced by an adequate increase in chlorophyll a, confirms that the higher LNP dosage has caused phytotoxic effects.

As for carotenoid and anthocyanin, Figure 4 shows the results found in the samples treated with the different doses of LNP compared to the controls. Carotenoids are essential light-harvesting pigments that act in photosynthesis, but they also show antioxidant activity. For this reason, they play a crucial role for plants in contributing to remove reactive oxygen species (ROS) [72]. In particular, carotenoids protect chloroplasts from ROS, quenching the chlorophyll on the singlet or triplet forms [73]. Our data show that T80 and T312 exerted a stimulatory action on the carotenoids biosynthesis. The increase in carotenoids found can be explained as a protective response to LNP treatments. The amount of carotenoids is also related to the content of chlorophyll a. Indeed, plants can perceive increases in the content of this pigment as a signal which, in turn, stimulates the carotenoid biosynthesis [72]. Based on these results, the use of non-excessive doses of LNP can be proposed as a strategy to raise the production of these beneficial substances.

As for anthocyanin, increases were generally observed in maize following the different treatments with LNP. Anthocyanin belongs to the flavonoid family, and they show fundamental properties in plants and therapeutic effects in humans [74]. Anthocyanin can act on ROS into the vacuole, inhibiting and contrasting the lipid peroxidation, and in general, their content can be increased in response to various environmental stresses [75]. In light of the above, maize perceived T80 and T312 as a signal at a non-phytotoxic level, increasing anthocyanin content. Indeed, anthocyanins are synthesized in the phenylpropanoid pathway [74], and their increase following the exposure to LNP can be explained by the capacity of substances containing phenols to induce the above metabolic pathway [64]. Moreover, the chlorophyll content can also be linked to anthocyanin increase, being the increase in chlorophyll content a possible physiological response that can compensate for eventual increases in anthocyanin [69]. At the highest dosages of LNP, the modest increase in anthocyanin resulted from the phytotoxic effects of the treatment, which hampered the plant ability to react to the stress.

The last biochemical parameter studied in response to LNP treatments was the total soluble protein content, as this is significantly related to a plant’s ability to assimilate nutrients, and it can also be indicative of phytotoxicity [36,76]. Only T80 increases the soluble protein content significantly compared to the control. This positive effect can be explained based on the beneficial role of phenols on maize seedlings. As already discussed, they can stimulate in treated plants the nitrogen content, which is essential for protein biosynthesis [64]. In the case of T5000, the reduction of soluble protein may be the consequence of the phytotoxic effects provoked by this treatment. In fact, this dosage probably interfered with the protein biosynthesis or caused oxidative stress leading to protein degradation [36]. Likewise, it has been documented that the toxic effects of high concentrations of lignin nanoparticles may be due to their capacity to diffuse into mitochondria or other cellular organelles, thus inhibiting critical cellular functions [8].

## 5. Conclusions

In this study, the effect of nanostructured lignin microparticles on a crop was investigated for the first time. Maize seeds primed with LNP showed interesting physiological and biochemical responses. In particular, the experimental findings suggest using nanostructured lignin microparticles at specific doses (T80 and T312) as a promising tool to stimulate positive biological responses in plants. On the other hand, higher dosages lost efficacy (T1250) or even resulted in phytotoxic effects (T5000 and T20000). As the LNP concentration increased, the beneficial effects were probably overwhelmed by too high concentrations of lignin constituent units (p-hydroxyphenyl, guaiacyl and syringyl). As reported in the literature, excessive amounts of these constituents can inhibit plant development [45]. However, given the opportunity offered by this biomass, further research is necessary to understand the nature of both the positive and negative effects. This aspect is of primary interest, also considering the large availability of this biomass, the disposal of which can impact the environment, and the importance of finding new biostimulating substances for agriculture.

## Figures and Tables

**Table 1:** Effects of the treatments with different concentrations of LNP on maize seed germination and radicle length (T80, T312, T1250, and T5000 refer to LNP concentration applied to seed for priming). The germination was recorded 4 days after the treatments, while the radicle length at 5 days.

	Germination (%)	Radicle Length (cm)
control	76.7 ^b^	1.95 ^b^
T80	83.3 ^ab^	3.27 ^a^
T312	90.0 ^a^	3.16 ^a^
T1250	96.7 ^a^	3.10 ^a^
T5000	90.0 ^a^	3.33 ^a^
T20000	56.7 ^c^	1.75 ^b^

Letters in the table, if different, indicate statistically significant differences for *p* < 0.05 between treatments.

## Data Availability

Data will be available on request to the corresponding author.
